# Dimensionality–Driven Electronic Modulation at Metal–Perovskite Interfaces for Electrochemical CO_2_ Reduction

**DOI:** 10.1002/smll.75118

**Published:** 2026-08-10

**Authors:** Seunghyun Chun, Hakhyeon Song, Myeongbum Ko, Yeneul Lim, Dongho Heo, Jae Won Shin, Hyunsik Kim, Hyeyoung Shin, Hyosun Lee

**Affiliations:** ^1^ Department of Materials Science and Engineering University of Seoul Seoul Republic of Korea; ^2^ George W. Woodruff School of Mechanical Engineering Georgia Institute of Technology Atlanta Georgia USA; ^3^ Graduate School of Energy Science and Technology (GEST) Chungnam National University Daejeon Republic of Korea; ^4^ Center For Advanced Reaction Dynamics Institute For Basic Science (IBS) Daejeon Republic of Korea; ^5^ Department of Chemistry College of Sciences Kyung Hee University Seoul Republic of Korea

**Keywords:** Au oxidation state, electrochemical CO_2_RR, metal–metal oxide interface, perovskites 2D nanosheet

## Abstract

Electrochemical CO_2_ reduction reaction (CO_2_RR) offers a sustainable approach for converting CO_2_ into value–added chemicals; however, its practical implementation remains limited by high overpotentials and poor selectivity. Metal–oxide interface engineering has recently emerged as a promising strategy to overcome these limitations. While the modulation of metal–support interactions is well established in gas–phase catalysis, their mechanistic manifestation within electrochemical interfaces remains insufficiently understood. Specifically, the influence of support dimensionality on the local CO_2_RR microenvironment has not been addressed. In this study, we systematically investigate the role of support dimensionality by comparing Au nanoparticles (NPs) supported on two‐dimensional (2D) Ca_2_Nb_3_O_10_ (CNO) and three–dimensional (3D) KCa_2_Nb_3_O_10_ (KCNO) perovskite oxides as well–defined model systems. The 2D Au/CNO catalyst demonstrated a marked enhancement in CO_2_RR performance, achieving a higher CO Faradaic efficiency (FE) than that of the 3D Au/KCNO catalyst. We revealed that the enhanced activity of Au/CNO is attributed to a higher proportion of partially oxidized Au species (Au^δ+^) and improved interfacial electronic polarization of Au on CNO. These findings underscore the importance of the oxide support structure in modulating electronic environments and metal–oxide interactions, providing new insights for the rational design of efficient CO_2_RR catalysts.

## Graphical abstract

Two–dimensional (2D) Ca_2_Nb_3_O_10_ (CNO) support enhances metal–oxide interactions in Au catalysts for the electrochemical CO_2_ reduction reaction (CO_2_RR). This promotes partially oxidized Au species, efficient interfacial charge transfer, and higher CO selectivity compared with three–dimensional (3D) KCa_2_Nb_3_O_10_. These findings highlight the critical role of perovskite oxide dimensionality in CO_2_RR catalyst design.

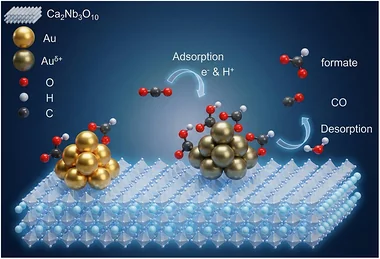

## 1 Introduction

The persistent reliance on fossil fuels has led to elevated atmospheric concentrations of carbon dioxide (CO_2_), driving major environmental consequences including climate change, rising sea levels, and ocean acidification [1, 2, 3, 4, 5]. Addressing these escalating challenges requires the development of sustainable strategies capable of simultaneously reducing CO_2_ emissions and producing clean energy. Among the various approaches, the electrochemical CO_2_ reduction reaction (CO_2_RR) has attracted considerable attention as a promising pathway for converting CO_2_ into value–added chemicals such as CO, HCOOH, CH_4_, C_2_H_4_, and C_2_H_5_OH [6, 7, 8]. The CO_2_RR progresses under ambient conditions, and the product selectivity can be modulated by adjusting the catalyst, electrolyte and electrolysis environments [9, 10, 11]. However, a substantial overpotential is required to activate the thermodynamically stable CO_2_ due to its strong C═O double bonds [12]. Therefore, the process is impeded by low energy efficiency and poor selectivity, primarily attributed to the competitive hydrogen evolution reaction (HER).

A wide range of metal and/or metal oxide catalysts have been explored for CO_2_RR, and considerable efforts have focused on enhancing their catalytic performance by tuning catalyst size, morphology, composition, and surface functionality [13, 14, 15, 16]. Recently, metal–oxide interfaces have emerged as a distinct class of active sites for CO_2_RR, offering unique local electronic structures that facilitate intermediate binding and accelerate reaction kinetics [17, 18, 19, 20, 21, 22, 23, 24, 25]. For example, Sun et al. demonstrated that Au nanoparticles (NPs) supported on CeO_2_ exhibited strong metal–support interaction (SMSI), which promoted charge transfer and significantly influenced the adsorption/desorption behavior of intermediates during CO_2_RR [26]. Due to its high CO selectivity and electrochemical stability, Au is widely used as a model catalyst for investigating metal–oxide interfaces during CO_2_RR. Similarly, CeO_2_–armored Ag nanowires show enhanced CO_2_–to–CO conversion due to electronic modification at the Ag–CeO_2_ interface, which lowers the *COOH formation energy and improves both activity and stability under high current densities [27]. While interest in such SMSI has continued to grow, systematic investigations on how the dimensionality of oxide supports, such as two–dimensional and three–dimensional structures, influences interfacial properties including charge transfer, intermediate binding, and product selectivity, remain limited. In this context, two–dimensional heterostructured architectures have been reported to facilitate charge transport to the surface and enhance interfacial charge separation in CO_2_ reduction systems. These findings suggest that structural dimensionality and interfacial architecture can play critical roles in modulating catalytic behavior [28].

Perovskite oxides, which have recently attracted attention as promising materials for CO_2_RR, offer a versatile platform for exploring the role of support dimensionality due to their flexible crystal chemistry and structural tunability [29, 30, 31]. The perovskite structures enable precise control over their electronic structure, surface properties, and interfacial behavior. Notably, certain layered perovskite derivatives such as Ca_2_Nb_3_O_10_ (CNO) can be exfoliated into atomically thin two–dimensional nanosheets, while maintaining a closely related three–dimensional counterpart such as KCa_2_Nb_3_O_10_ (KCNO) [32, 33, 34, 35, 36]. This creates ideal model systems for directly comparing the impact of support geometry on catalytic performance under otherwise similar chemical compositions. Moreover, structural stability, high surface area, and tunable electronic environments collectively provide a strong basis for utilizing oxide supports in mechanistic studies of metal–oxide interactions in CO_2_RR.

In this study, we investigate the relationship between Au and a series of perovskite oxide–based materials, exfoliated two–dimensional CNO and three–dimensional K–intercalated KCNO to gain fundamental insights into how support structures influence CO_2_RR activity and catalytic properties. By incorporating Au NPs onto each support, we fabricated well–defined Au–decorated CNO (Au/CNO) and KCNO (Au/KCNO) as model systems. The two–dimensional Au/CNO exhibited superior CO production, achieving a CO Faradaic efficiency (FE) of 63.5% at −0.85 V_RHE_, which is 8% higher than that of the three–dimensional Au/KCNO. X–ray photoelectron spectroscopy (XPS) reveals that Au/CNO stabilizes a higher fraction of Au^δ+^ species, consistent with strengthened binding energy of key intermediates for CO_2_RR. Furthermore, the density functional theory (DFT) calculations indicate stronger interfacial electronic polarization and greater electron depletion of Au at the Au/CNO interface compared to Au/KCNO. These findings establish a clear connection between support dimensionality and interfacial electronic structure, elucidating how metal–oxide interactions govern the local reaction environment and CO_2_RR selectivity, and offering guidance for the rational design of efficient catalysts.

## 2 Results and Discussion

The CNO nanosheet was synthesized through a multi‐step process, as shown in Figure 1a. First, the bulk perovskite KCNO was prepared by ball–milling following by calcination. Second, a large organic cation, such as tetrabutylammonium hydroxide (TBAOH), was added into the KCNO solution, inducing exfoliation of individual CNO layers because of the expansion of the interlayer spacing (see the Supporting Information for further details) [37]. A CNO monolayer consists of three NbO_6_ layers with Ca ions located in voids between NbO_6_ layers. Each NbO_6_ unit is composed of a Nb atom with six oxygen atoms, forming an octahedral structure. To investigate the morphology of KCNO and CNO, scanning electron microscopy (SEM) and transmission electron microscopy (TEM) analyses were conducted (Figure 1b–g). As shown in Figure 1b,c, SEM and TEM images reveal that KCNO exhibits a morphology closer to 3D bulk structures rather than 2D NS. On the other hand, CNO appears as a thin, transparent NS, as evidenced by the visibility of overlapping layers, indicating an ultrathin morphology (Figure 1e,f). The TEM measurements revealed a thickness of 1.44 ∼ 1.46 nm (Figure S2). According to the CNO unit cell, the total length of three NbO_6_ layers with Ca atoms is nearly identical to the measured thickness [38, 39]. High–resolution TEM (HRTEM) images reveal the lattice spacing of 0.29 and 0.39 nm, corresponding to the (110) plane of KCNO and (100) plane of CNO (Figure 1d,g). To further confirm the crystalline structure, X–ray diffraction (XRD) analysis was carried out, showing strong peaks at 5.9°, 23.1°, 29.4°, 31.3°, and 32.8°, which are assigned to the (020), (110), (062), (170), and (112) planes of orthorhombic KCNO (JCPDS 98–009–1098) (Figure 1h). After exfoliation, the CNO nanosheets showed diffraction peaks at 23.5°, 32.9°, and 47.1°, corresponding to the (020), (220), and (040) planes, respectively (Figure 1i) [40]. These peaks indicate that the crystalline Ca_2_Nb_3_O_10_ framework was retained even after exfoliation into nanosheets. In addition, peaks at 5.9°, 11.7°, 17.6°, and 41.9°, which are located close to the (020), (040), (060), and (0 14 0) reflections of orthorhombic KCNO, respectively, suggest the presence of a small fraction of residual KCNO–like layered domains in the CNO support.

**FIGURE 1:**
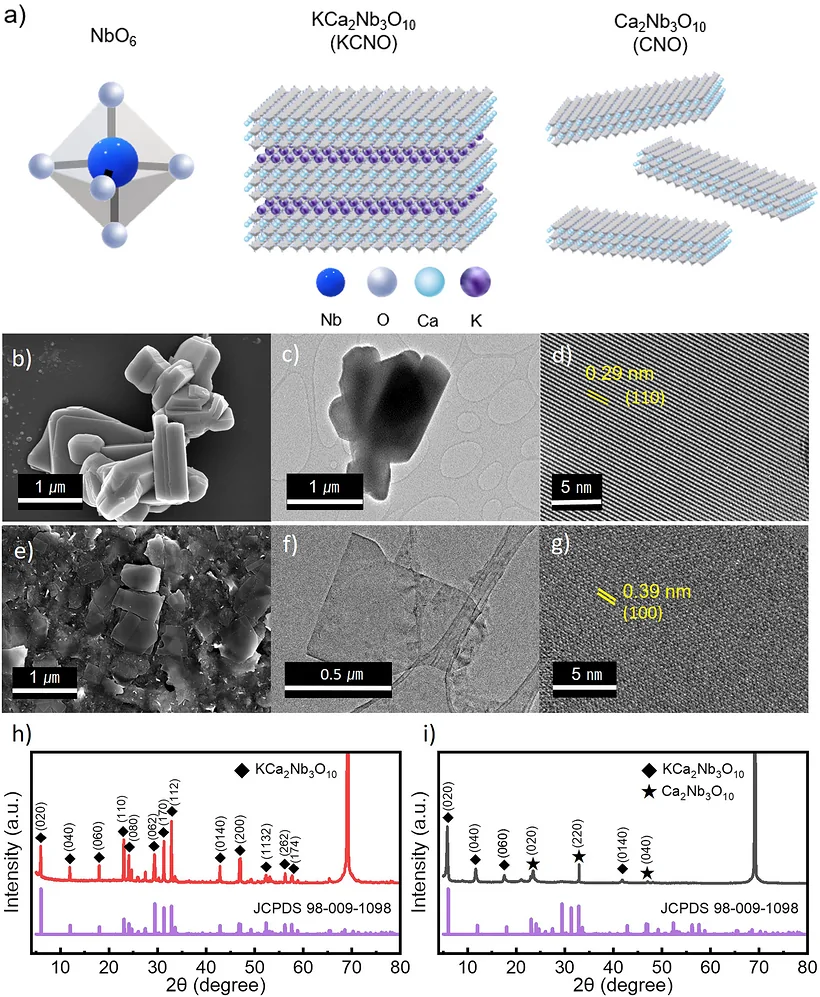
(a) Schematic of the NbO_6_ octahedra, bulk KCa_2_Nb_3_O_10_ (KCNO) and Ca_2_Nb_3_O_10_ (CNO) nanosheet. (b) SEM, (c) TEM, and (d) HRTEM images of KCNO, showing the layered perovskite oxide morphology and high crystallinity; (e) SEM, (f) TEM, and (g) HRTEM images of CNO, revealing the ultrathin nanosheet structure with clearly resolved lattice planes. The XRD patterns for (h) KCNO and (i) CNO. The reference diffraction peaks of KCNO are indexed according to JCPDS card No. 98–009–1098.

Au–deposited KCNO (Au/KCNO) and CNO (Au/CNO) catalysts were synthesized to enable a comparative investigation of the structures, and properties. Inductively coupled plasma mass spectrometry (ICP–MS) analysis confirmed that the actual Au content of Au/KCNO and Au/CNO were 10.45 wt.% and 11.75 wt.%, respectively (Table S1). These catalysts are denoted as Au/KCNO and Au/CNO throughout the manuscript unless the Au loading is specifically discussed. In Figure 2a,b, TEM was used to confirm the morphology of Au/KCNO and Au/CNO, and HRTEM was employed to examine the crystallinity of Au NPs on the catalyst surface. In the Au/KCNO sample, lattice fringes with spacings of 0.23 nm were observed, corresponding to the (111) planes of metallic Au, respectively (Figure 2a). Similarly, the Au/CNO sample exhibited a lattice spacing of 0.23 nm, also assigned to the (111) plane of Au (Figure 2b) [41, 42, 43]. These observations confirm the successful deposition of crystalline Au NPs on both KCNO and CNO supports. The average Au NP sizes were determined to be 33.3 ± 11.7 nm for Au/KCNO and 43.7 ± 16.4 nm for Au/CNO, indicating that both catalysts contain polydisperse Au NPs with comparable size distributions (Figure S3a,b). Although Au/CNO had a slightly higher Au loading than Au/KCNO, its larger average Au particle size suggests that it does not possess a larger exposed Au surface area. A simple geometric estimation based on the ICP–MS–derived Au loadings and TEM–derived particle sizes indicates that the relative geometric Au surface area of 0.86 for Au/CNO compared with Au/KCNO, suggesting that Au/CNO has a slightly lower exposed Au surface area despite its higher Au loading. XPS was conducted to investigate the surface chemical states of Au–loaded catalysts. As shown in Figure 2c,f, both Au/KCNO and Au/CNO samples exhibit characteristic Au^0^ peaks at binding energies of approximately 84.0 eV (Au 4f_7/2_) and 87.7 eV (Au 4f_5/2_) [44]. In addition to metallic Au^0^, oxidized Au species (Au^+^ and Au^3+^) were also observed at higher binding energies adjacent to the Au^0^ peaks. The relative peak area ratio of oxidized Au species was found to be 15.9% for Au/CNO and 8.3% for Au/KCNO, indicating a clear support–dependent difference. Figure 2d presents the K 2p spectra of Au/KCNO, where distinct peaks corresponding to K^+^ were observed at 293.0 eV (K 2p_3/2_) and 295.8 eV (K 2p_1/2_), indicative of the presence of interlayer potassium ions [41]. In contrast, the K signal was significantly diminished in the Au/CNO, consistent with the exfoliation processes employed during CNO synthesis (Figure 2g). Additionally, a broad peak centered at approximately 292.1 eV was observed in both samples, which is attributed to CF_2_ groups originating from the Nafion 117 binder used in the fabrication of working electrodes, partially overlapping with the K 2p signal (Figure 2d,g). As shown in Figure 2e,h, the Nb 3d spectra for both supports exhibited dominant Nb^5+^ peaks at approximately 207.6 eV (Nb 3d_5/2_) and 210.4 eV (Nb 3d_3/2_), accompanied by minor components at slightly lower binding energies that were attributed to Nb^4+^ species [42]. The presence of Nb^4+^ is interpreted as a result of localized surface reduction, likely arising from local electronic interactions during catalyst synthesis or gold deposition. No substantial difference was observed in the spectral shape or oxidation state distribution of Nb between the CNO and KCNO supports, indicating that the interlayer potassium ions do not significantly affect the Nb oxidation environment. To further exclude the possible influence of the Nafion 117 binder, XPS measurements were additionally performed on binder–free powder samples of Au/KCNO and Au/CNO (Figure S4). The powder XPS spectra showed the disappearance of the Nafion–derived CF_2_ peak, while the characteristic signals and chemical states of the catalyst components were retained. These results support that the observed surface chemical states mainly originate from the catalyst materials themselves.

**FIGURE 2:**
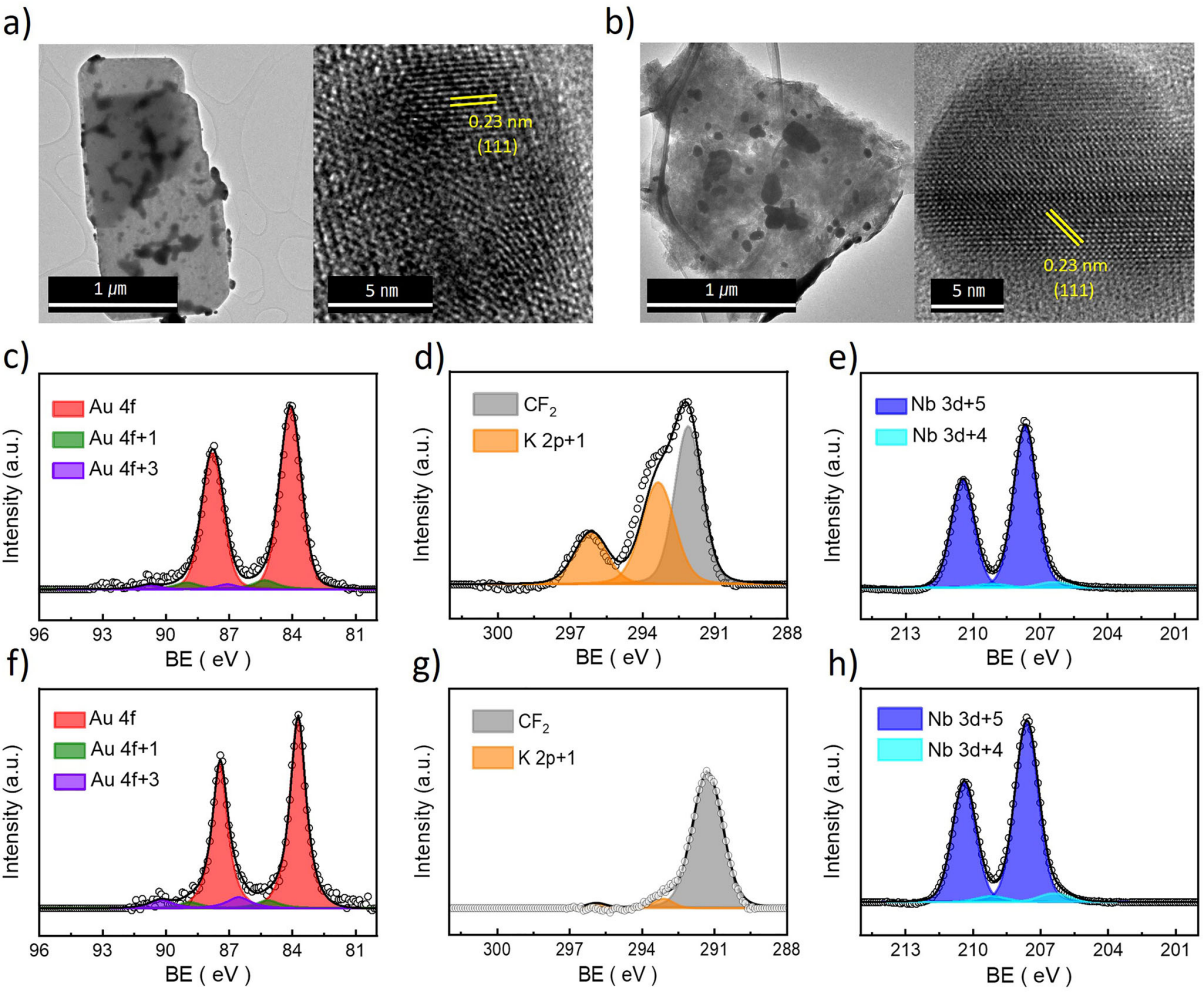
(a) The TEM images of Au–loaded KCNO (Au/KCNO) and HRTEM images of Au NPs on Au/KCNO. (b) The TEM images of Au–loaded CNO (Au/CNO) and HRTEM images of Au NPs on Au/CNO. XPS spectra data of (c) Au 4f, (d) K 2p and (e) Nb 3d for Au/KCNO, and (f) Au 4f, (g) K 2p and (h) Nb 3d for Au/CNO.

In Figure 3, we evaluated the electrochemical properties of CNO and KCNO perovskite metal oxide materials using a custom–designed gas–diffusion electrode reactor (see the experimental methods in Supporting Information for further description). All electrochemical potentials reported in this work were iR–corrected using the uncompensated resistance obtained from PEIS measurements. The geometric current densities for bare CNO and KCNO appeared nearly identical over the potential range from −0.45 to −1.05 V_RHE_, indicating similar overall electrochemical behavior (Figure S5). These results suggest that the incorporation of K^+^ into the CNO structure, which induces a transition from a two–dimensional to a three–dimensional morphology, does not significantly affect the electrochemical performance when normalized by geometric area. However, the current densities normalized by the electrochemically active surface area (ECSA) revealed structure–dependent intrinsic activity (Figure 3a and Figure S6). As shown in Figure 3a, CNO consistently exhibited higher ECSA–normalized current densities than KCNO across the entire potential range, indicating superior intrinsic activity. For example, at each potential, CNO achieves approximately 1.3–1.5 times higher current density than KCNO. Correspondingly, ECSA measurements exhibited that KCNO possesses an ECSA approximately 1.7 times larger than that of CNO, likely due to structural changes induced by K^+^ intercalation (Figure S6). Despite this increase in surface area, the lower intrinsic activity of KCNO highlights fundamental differences in electrocatalytic behavior between the two materials.

**FIGURE 3:**
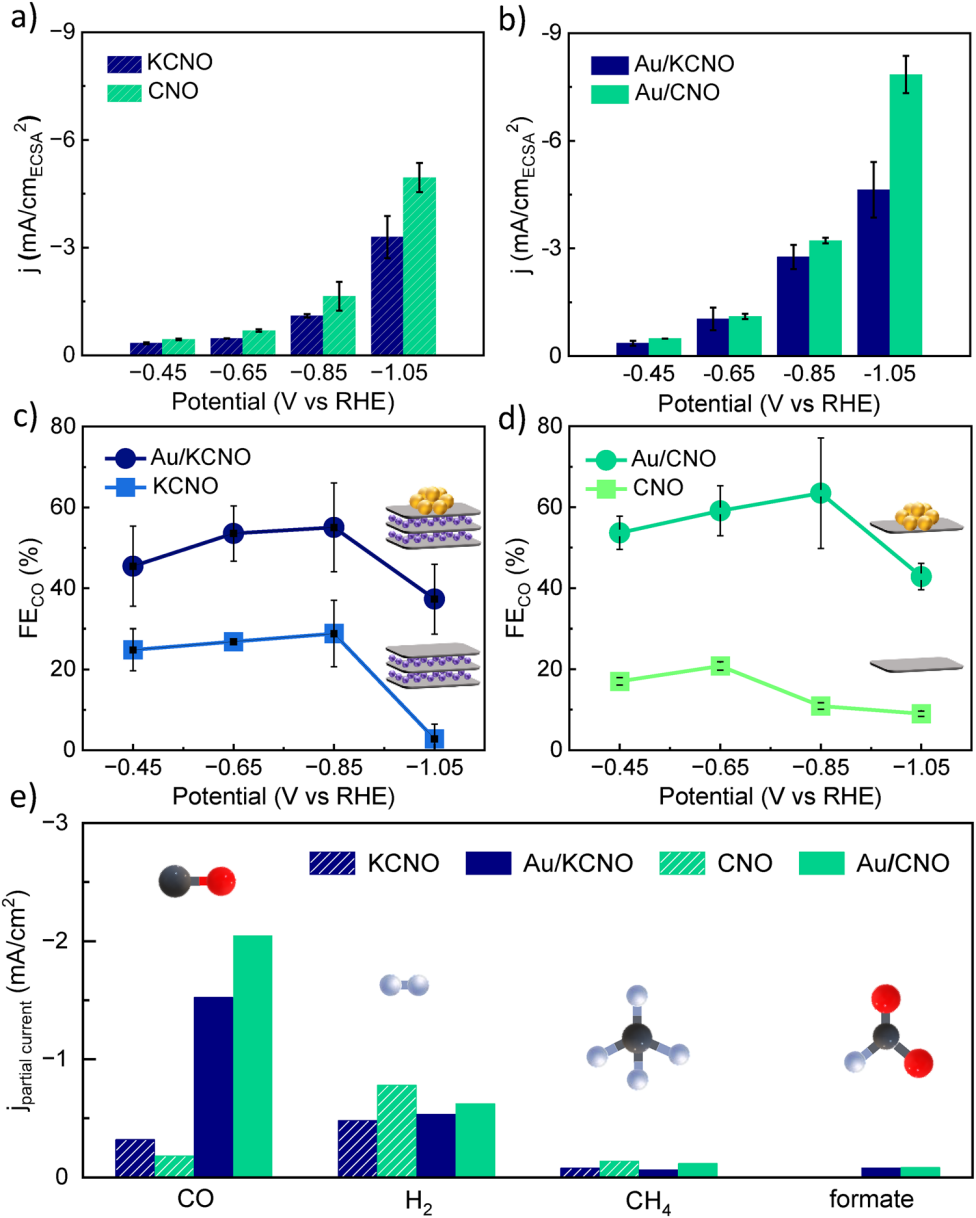
Normalized current density by ECSA for (a) bare CNO and bare KCNO and (b) Au/KCNO and Au/CNO. Faradaic efficiency of CO as a function of potential for (c) KCNO and Au/KCNO, and (d) CNO and Au/CNO. (e) Partial current densities of CO, H_2_, CH_4_, and formate at −0.85 V_RHE_ for KCNO, CNO, Au/KCNO, and Au/CNO.

Upon Au decoration, both CNO and KCNO catalysts exhibit a significant increase in ECSA–normalized current densities, as shown in Figure 3b. Compared to their respective bare forms, Au/CNO and Au/KCNO show approximately 1.1– to 1.7–fold enhancement across the entire potential range, indicating improved intrinsic activity. Notably, the two–dimensional Au/CNO consistently outperforms the three–dimensional Au/KCNO at all applied potentials from −0.45 to −1.05 V_RHE_. At low overpotentials (e.g., −0.45 V_RHE_), both materials exhibit relatively modest activity, with Au/CNO already displaying slightly higher performance. As the potential becomes more negative from −0.45 to −1.05 V_RHE_, the current density of Au/CNO increases more rapidly than that of Au/KCNO, leading to a substantial gap in performance at −1.05 V_RHE_. At −1.05 V_RHE_, Au/KCNO reaches a current density of −4.6 mA/cm^2^, whereas Au/CNO achieves a significantly higher value of −7.8 mA/cm^2^, underscoring the more favorable catalytic environment provided by the CNO support and the stronger Au–oxide interaction. In contrast, the geometric area–normalized current densities show a different trend (Figure S6). From lower to moderate potentials (−0.45 to −0.85 V_RHE_), Au/KCNO exhibits slightly higher geometric current densities than Au/CNO, possibly due to its larger surface area. However, this trend reverses at higher overpotentials (−1.05 V_RHE_), where Au/CNO surpasses Au/KCNO, aligning with the intrinsic activity trends observed in the ECSA–normalized results. This potential–dependent crossover highlights the importance of evaluating both intrinsic and apparent activities to fully understand catalytic behavior.

Au deposition on CNO and KCNO supports modulates not only the current densities but also the product selectivity toward CO formation. For KCNO, CO FE remained around 20% within the potential range from −0.45 to −0.85 V_RHE_. However, when the potential is negatively increased to −1.05 V_RHE_, the CO FE becomes negligible (Figure 3c). CNO exhibits a similar trend, showing relatively lower CO FE (10% – 20%) in the same potential range (Figure 3d). Notably at −1.05 V_RHE_, the CO FE for CNO remains at 10%, which is higher than that of KCNO. It is also noted that the total FE of both CNO and KCNO does not reach 100% (Figure S7a,b). The FE measurements were reproducible across independent experiments, suggesting that the observed discrepancy is unlikely to arise solely from experimental uncertainty. While the exact origin of the missing FE remains unclear, a fraction of the charge may be associated with non–Faradaic processes commonly observed in oxide–based electrodes, such as surface charging and capacitive contributions [43]. In contrast, the introduction of Au results in significantly improved total FE values, approaching 100% (Figure S7c,d). This suggests that the synergistic interaction between Au and the perovskite supports promotes more efficient Faradaic conversion pathways toward CO_2_RR.

The incorporation of Au NPs significantly enhances CO selectivity in both CNO and KCNO systems (Figure 3c,d). Notably, the CO FE of Au/CNO is consistently higher than that of Au/KCNO throughout the entire potential range. At −0.85 V_RHE_, Au/CNO achieved a CO FE of approximately 63.5%, representing a 5.8–fold increase compared to its pristine counterpart (10.9%). In contrast, Au/KCNO reached a CO FE of ∼55% at the same potential, corresponding to a 1.9–fold enhancement relative to bare KCNO. Interestingly, Au/CNO exhibits a higher CO FE despite having a larger average Au NP size and a slightly lower estimated geometric Au surface area than Au/KCNO. This observation suggests that, despite comparable Au loadings, the interfacial environment between Au NPs and the CNO nanosheet support contributes predominantly to the enhanced CO formation, outweighing the possible effects related to Au particle size and exposed surface area in the present catalyst system. We further analyzed the partial current densities for individual products to better capture the intrinsic reaction kinetics of CO_2_RR (Figure 3e). Unlike FE, which merely describes the product distribution, the partial current density directly correlates with the rate of product formation, offering a quantitative and time–resolved measure of catalytic activity [44, 45, 46, 47]. It is worth noting that the CO partial current density (j_CO_) is markedly enhanced upon Au decoration, with Au/CNO achieving j_CO_ of −2.0 mA/cm^2^ at −0.85 V_RHE_. This reflects a significant improvement in the CO formation rate, exceeding that of bare CNO (−0.17 mA/cm^2^) by more than 11.4–fold. Similarly, Au/KCNO shows a substantial increase in j_CO_ (∼−1.5 mA/cm^2^). This enhancement aligns with previous works suggesting that combining metals with metal oxide supports can result in superior CO_2_RR performance compared to either component alone [48, 49, 50, 51, 52, 53, 54, 55]. In contrast, the H_2_ partial current densities of CNO, KCNO, Au/CNO, and Au/KCNO show no appreciable difference across all samples, indicating that the hydrogen evolution rate is largely unaffected by either Au decoration or the nature of the oxide support. This finding supports that the Au–perovskite system selectively enhances CO formation without altering HER kinetics, thereby steering the reaction pathway toward CO production rather than indiscriminately increasing total activity. Additionally, to evaluate the effect of Au loading, we compared CO_2_RR performance at two different Au contents: 2.62 wt.% and 11.75 wt.%. TEM and XPS analyses confirmed the successful deposition of Au NPs on CNO nanosheets and the surface chemical states of Au_2.62wt%_/CNO, respectively (Figures S8 and S9). ECSA for Au_2.62wt%_/CNO exhibited between bare CNO and Au_11.75wt%_/CNO (Figure S10). The influence of Au content was evident in both the ECSA normalized current densities and CO FE at all applied potentials (Figure S11). As shown in Figure S12, increasing the Au content on CNO leads to a progressive enhancement in CO FE, indicating that higher Au deposition may contribute to the formation of more active and selective catalytic sites for CO_2_–to–CO conversion.

Trace amounts of CH_4_ and formate were also detected (Figure 3e). The formation of CH_4_ was observed across all tested catalysts, including bare supports (KCNO and CNO) as well as Au–loaded samples (Au/KCNO and Au/CNO). Notably, CH_4_ production remains nearly equivalent irrespective of the presence of Au. Moreover, CH_4_ FE gradually decreased at more negative potentials, indicating that the reaction pathway toward CH_4_ is suppressed at higher overpotential (Figure S7). In contrast, formate was exclusively detected in Au–containing catalysts, while bare supports produced no measurable formate (Figure S7). This suggests that interface between Au and perovskite oxides may promote C─H bond formation. However, the formation of formate proceeds via early C─H bond formation, while methane formation requires stepwise hydrogenation of CO–derived intermediates. This mechanistic distinction highlights why Au–based catalysts favor formate generation with limited CH_4_ production, as the weak binding of CO on Au surfaces prevents the deep reduction steps necessary for methane formation. Consistently, a previous study has reported that Au can generate formate as a minor product [56]. The observation of formate in both Au/KCNO and Au/CNO samples thus supports the intrinsic role of Au in CO_2_RR in this system. These findings highlight the dual influence of both the Au active sites and the metal–metal oxide interface in determining the overall product formation, even in the minor product regime.

To confirm that the CO_2_RR performance discussed above was evaluated before significant performance decay, chronopotentiometric stability measurements were conducted for 180 min under a constant current condition that maintained the potential at approximately −0.85 V vs RHE (Figure S13). During the 180 min electrolysis, both catalysts maintained relatively stable potentials, while their CO selectivity showed different stability windows: Au/KCNO maintained a stable CO FE for approximately 70 min, whereas Au/CNO retained stable CO selectivity for approximately 130 min. These results indicate that Au/CNO provides improved operational stability for CO production compared with Au/KCNO under the tested conditions. Importantly, all CO_2_RR performance data discussed above were collected within the first 1 h of electrolysis. For all measurements presented with error bars, two independent measurement sets were performed under identical conditions using the same type of catalyst sample. The error bars represent the standard deviation of the two independent measurements. Therefore, the activity and selectivity trends discussed in Figure 3 were obtained before significant time–dependent degradation occurred, indicating that the comparative CO_2_RR performance was not substantially affected by catalyst deactivation during the measurement. To further examine the catalyst state after under conditions consistent with the CO_2_RR performance evaluation, post–reaction XRD and SEM analyses were performed after 1 h of electrolysis. Comparison of the XRD patterns and SEM images before and after CO_2_RR revealed no discernible changes in either the phase structure or the overall morphology of the Au/CNO and Au/KCNO catalysts (Figures S14 and S15), suggesting that both catalysts largely retained their structural integrity during the measurement window.

To further understand how different perovskite supports influence the electronic interaction with Au nanoparticles, we investigated the interfacial charge–transfer properties of the catalysts using electrochemical impedance spectroscopy (EIS), XPS, and DFT calculations. Incorporating a metal component into a metal oxide can significantly alter its electronic properties, including charge transfer resistance (*R*_ct_), due to the enhanced conductivity and interfacial charge mobility provided by the metallic sites. As shown in the EIS data, bare KCNO exhibits a larger semicircle than Au/KCNO (Figure 4a). Equivalent circuit fitting reveals that the *R*_ct_ of KCNO is approximately 3.33 Ω, while that of Au/KCNO is slightly reduced to 3.21 Ω, indicating a modest improvement in charge transfer in the presence of Au. In contrast, a more pronounced decrease in *R*_ct_ is observed in the CNO–based system. As shown in Figure 4b, the *R*_ct_ of bare CNO is 3.57 Ω, which significantly decreases to 3.02 Ω with Au, representing an approximately 15% reduction, compared to only 4% in the KCNO system. This notable difference highlights that the interaction between Au and the CNO support more effectively facilitates interfacial electron transfer than in the KCNO system. This improved charge transport in the metal–metal oxide is consistent with the significantly higher current densities observed in Figure 3a,b, where both Au/CNO and Au/KCNO exhibit markedly enhanced overall activity compared to their bare counterparts.

**FIGURE 4:**
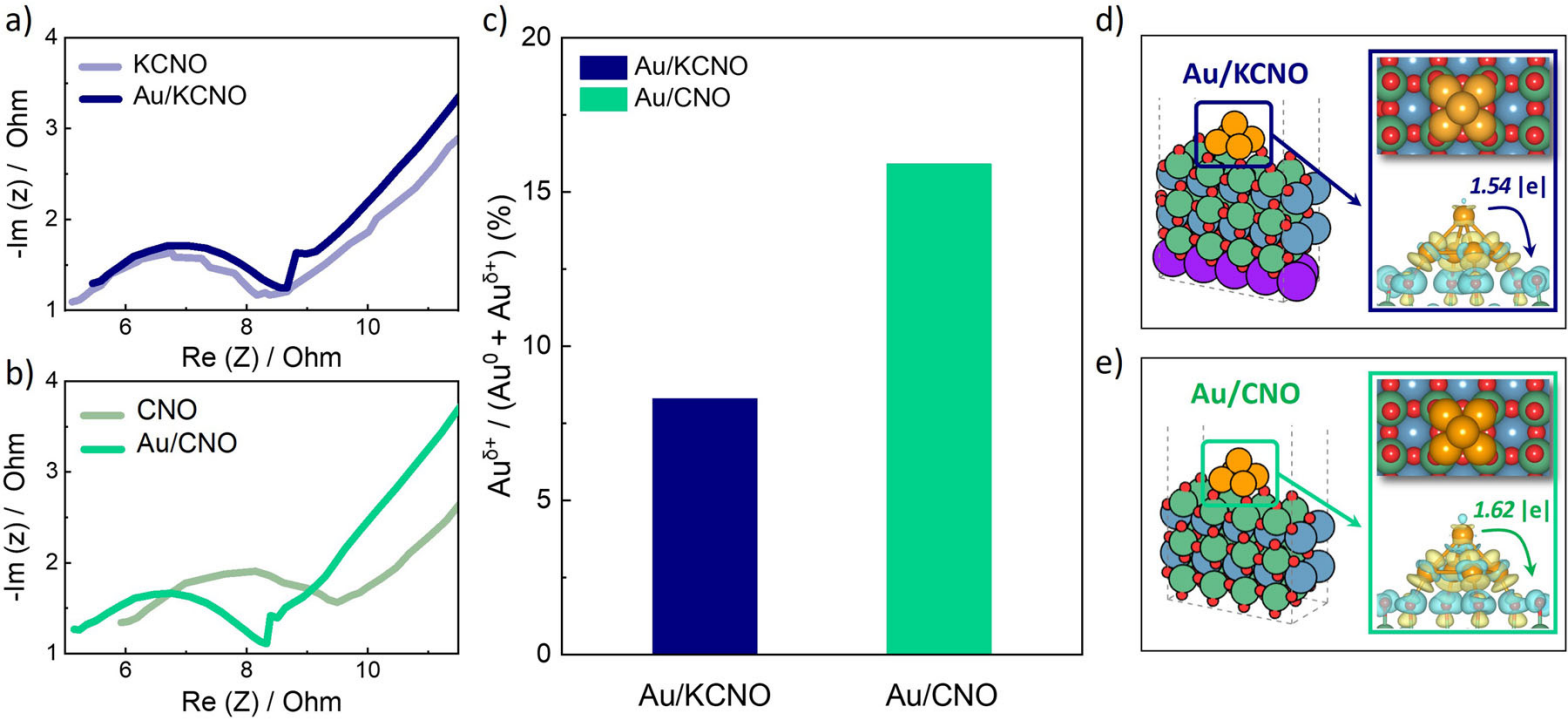
Nyquist plots of (a) KCNO and Au/KCNO and (b) CNO and Au/CNO. (c) The ratio of $Au^{\delta^{+}}$ for Au/KCNO and Au/CNO, calculated from the Au 4f XPS spectra. (d) Charge density difference map of Au/KCNO and (e) Au/CNO, visualized at an isosurface level of 0.005 *e*/Bohr^3^. Yellow regions indicate charge accumulation, whereas blue regions denote charge depletion.

However, it is challenging to fully rationalize the observed product selectivity solely based on the *R*_ct_, as the product distribution can also be strongly influenced by the binding energies of key intermediates, the oxidation states of the active species, and the local microenvironment [57, 58, 59]. Among these factors, the control of oxidation states in metal–metal oxide catalysts plays a pivotal role in steering CO_2_RR selectivity [60, 61]. While metallic catalysts generally remain in the metallic state under reductive conditions, Au species in Au–metal oxide can exhibit partially oxidized states (Au^δ+^), likely due to strong interfacial interactions with the oxide support. Previous studies have suggested that partially oxidized Au species can influence the adsorption behavior of key CO_2_RR intermediates, including *COOH, thereby affecting catalytic selectivity [26, 62]. XPS confirms the presence of oxidized Au species in both Au/CNO and Au/KCNO (Figure 4c). Notably, the proportion of Au^δ+^ relative to the total Au species (Au^0^ + Au^δ+^) is substantially higher for Au/CNO (15.9%) than for Au/KCNO (8.3%), suggesting stronger support–induced electronic polarization of Au species in the CNO–supported catalyst.

To gain atomistic insight into the interfacial electronic structure responsible for the support–dependent Au oxidation behavior, density functional theory (DFT) calculations were performed with a focus on charge redistribution at the Au–oxide interface. Detailed computational models and methods are provided in the Supporting Information. Charge density difference (CDD) analysis reveals pronounced interfacial charge polarization in both systems, characterized by electron depletion localized on the Au cluster and corresponding charge accumulation at the Au–oxide interface. This result indicates strong electronic coupling between Au nanoparticles and the perovskite support, leading to support–dependent modulation of the electronic state of Au. Notably, the Au/CNO interface exhibits more extensive charge redistribution compared to Au/KCNO, indicating stronger electronic interaction between Au and the CNO support. This trend is quantitatively supported by Bader charge analysis [63], which yields cumulative positive charges of +1.54 |*e*| for Au/KCNO and +1.62 |*e*| for Au/CNO (Figure 4d,e), reflecting greater electron withdrawal from Au when supported on CNO. Together, the spatially resolved CDD visualization and quantitative Bader analysis demonstrate stronger interfacial electronic coupling in Au/CNO than in Au/KCNO, providing an atomistic basis for the experimentally observed higher fraction of Au^δ+^ species in Au/CNO.

To probe the catalytic role of the experimentally observed Au^δ+^ species, free–energy calculations were carried out for the CO_2_RR pathway by comparing the Au/CNO interface with a metallic Au(111) surface (Figure S18) [64]. Compared with the metallic Au surface, the Au/CNO interface exhibits more favorable adsorption of the key COOH intermediate, with the adsorption free energy decreasing from 0.703 to 0.568 eV. A similar trend is also observed for CO adsorption, resulting in a lower thermodynamic overpotential for overall reaction on Au/CNO. These results suggest that the electronically polarized Au environment at the Au/CNO interface facilitates CO_2_–to–CO conversion through enhanced stabilization of key CO_2_RR intermediates.

This difference may be associated with the distinct oxide morphologies of CNO and KCNO, as variations in surface structure can influence the interfacial coordination environment and electronic interactions with supported Au species. In Cu–CeO_2_ systems, for example, oxide supports with different geometries such as nanocubes, nanorods, and octahedra have been shown to modulate the stabilization of oxidation states and affect CO_2_RR selectivity, likely by altering the distribution of exposed crystal facets and the local electronic structure at the metal–oxide interface [65]. Earlier investigations revealed that the electronic structure of Au is influenced by surface hydroxyl groups [66]. As demonstrated herein, deposition of Au on hydroxylated and non–hydroxylated oxide surfaces resulted in a higher fraction of oxidized Au species on the hydroxylated sample. This observation suggests that OH groups are not merely passive surface ligands but actively interact with Au to generate oxidized species, thereby playing a crucial role in modulating its oxidation state. Furthermore, this finding corroborates our results in Figure S19, which show that the CNO supported catalyst possesses a higher density of OH groups and, correspondingly, a greater proportion of oxidized Au states. Importantly, the stability of the Au chemical states under CO_2_RR conditions, the Au 4f_7/2_ peak area ratios of Au^0^, Au^+^, Au^3+^ were compared before and after 1 h of electrolysis for all catalysts, including Au_2.62 wt.%_/CNO, Au_11.75 wt.%_/CNO, Au_10.45 wt.%_/KCNO (Figure S20 and Table S2). Although the Au content of Au/CNO is slightly higher than that of Au/KCNO, this modest difference alone is unlikely to fully account for the distinct CO_2_RR performance. Instead, the higher density of surface OH groups and the larger proportion of oxidized Au species in Au/CNO suggest that the CNO support plays an important role in modulating the surface chemical state of Au and promoting CO formation. As a result, Au/CNO exhibited a 52.6% higher CO FE and a 11.4–fold increase in CO partial current density compared to bare CNO, highlighting its enhanced catalytic performance (Figure 3d,e). These findings underscore the importance of tailoring perovskite structures to modulate interfacial properties such as *R*_ct_ and Au oxidation states, which collectively determine the catalytic efficiency and product selectivity of CO_2_ reduction.

## 3 Conclusions

In this study, we systematically investigated the influence of oxide support dimensionality on the electrocatalytic performance of CO_2_ reduction reaction by comparing two perovskite–based systems: exfoliated two–dimensional Ca_2_Nb_3_O_10_ (CNO) and three–dimensional K–intercalated KCa_2_Nb_3_O_10_ (KCNO), both decorated with Au NPs. Our results demonstrate that the dimensional structure of the oxide support plays a decisive role in modulating metal–oxide interfacial properties, electron transfer behavior, and ultimately the product selectivity and activity of CO_2_RR. The two–dimensional Au/CNO catalyst exhibited enhanced CO production performance compared to its three–dimensional counterpart Au/KCNO, achieving a CO FE of 63.5% at −0.85 V_RHE_, which is ∼8% higher than Au/KCNO. Additionally, CO partial current density was more than 11.4 times greater than that of bare CNO, confirming a strong effect of the Au/CNO interface. XPS analysis revealed a significantly higher proportion of oxidized Au species (Au^δ+^) on the CNO support, which is attributed to stronger metal–oxide interaction and is believed to enhance the binding of reaction intermediates (*COOH, *CO) crucial for CO formation. EIS further confirmed that the Au/CNO system exhibited a markedly lower charge transfer resistance (*R*_ct_) compared to Au/KCNO, reinforcing the notion that dimensionality directly influences the efficiency of interfacial electron transport. To further interpret the experimental observations at the atomic scale, DFT calculations were conducted to investigate interfacial charge redistribution. The results revealed stronger electronic polarization and greater electron depletion of Au at the Au/CNO interface compared to Au/KCNO, consistent with the higher fraction of partially oxidized Au species observed experimentally. These findings highlight the critical importance of oxide support and its interaction with Au NPs in determining CO_2_RR performance. The study underscores the potential of 2D perovskite nanosheets not only as structural supports but also as active contributors to charge redistribution and intermediate stabilization. This work provides valuable insights for the rational design of next–generation electrocatalysts, where careful engineering of support dimensionality and metal–oxide interactions can lead to enhanced selectivity, activity, and durability in CO_2_ conversion applications.

## Conflicts of Interest

The authors declare no conflicts of interest.

## Supporting information




**Supporting File**: smll75118‐sup‐0001‐SuppMat.docx.

## Data Availability

The data that support the findings of this study are available from the corresponding author upon reasonable request.
