# Supplementary material for: Dimensionality–Driven Electronic Modulation at Metal–Perovskite Interfaces for Electrochemical CO2 Reduction
Source: Small. 2026 Aug 10;22(57):e75118. doi: 10.1002/smll.75118 (PMC13659783; doi:10.1002/smll.75118)
Supplement: Supplementary file 1 [file SMLL-22-e75118-s001.docx]

*Supporting Information*

**Dimensionality-Driven Electronic Modulation at Metal- Perovskite Interfaces for Electrochemical CO_2_ Reduction**

Seunghyun Chun^1^^‡^, Hakhyeon Song^2‡^, Myeongbum Ko^3‡^, Yeneul Lim^1‡^, Dongho Heo^1^, Jae Won Shin^4^, Hyunsik Kim^1^*, Hyeyoung Shin^5^* and Hyosun Lee^1^*

^1^*Department of Materials Science and Engineering, University of Seoul, Seoul 02504, Republic of Korea*

^2^*George W. Woodruff School of Mechanical Engineering, Georgia Institute of Technology, Atlanta, GA 30332, USA*

^3^*Graduate School of Energy Science and Technology (GEST), Chungnam National University, Daejeon 34134, Republic of Korea*

^4^*Center for Advanced Reaction Dynamics, Institute for Basic Science (IBS), Daejeon 34141, Republic of Korea*

^5^*Department of Chemistry, College of Sciences, Kyung Hee University, Seoul 02447, Republic of Korea*

*Correspondence to: hyunsik.kim@uos.ac.kr, shinhy@khu.ac.kr, hyosunlee@uos.ac.kr

This file includes:

Experimental methods

Figure S1-S20

Table S1-S2

**Experimental methods**

**Materials.** Potassium carbonate (K_2_CO_3_), calcium carbonate (CaCO_3_), niobium pentoxide (Nb_2_O_5_), tetrabutylammonium hydroxide (TBAOH, 40 wt%), chloroauric acid (HAuCl_4_·xH_2_O), sodium citrate, and Nafion 117 solution were purchased from Sigma-Aldrich and used without further purification. Ethanol (EtOH) and deionized (DI) water were used as solvents throughout the synthesis.

**Synthesis of catalysts.** K_2_CO_3_, CaCO_3_, and Nb_2_O_5_ were mixed in stoichiometric ratios and ball-milled with zirconia balls in a polypropylene jar for 20 h. The resulting powder was dried and calcined in air at 1200 °C for 10 h. Proton-exchanged HCa_2_Nb_3_O_10_ (HCNO) was prepared by stirring 5 g of the calcined powder in 200 mL of 7 M HNO_3_ for 5 days. For exfoliation, 0.8 g of HCNO was added to 200 mL of DI water containing 1.02 mL of 40 wt% TBAOH and stirred for 7 days. The resulting dispersion was centrifuged at 2000 rpm for 10 min, and the CNO nanosheets was collected.

**Characterization.** Surface chemical states were characterized by X-ray photoelectron spectroscopy (XPS, Nexsa, Thermo Fisher), where Au, K, Ca, Nb, O, and C were each scanned 10 times, and Au was scanned 20 times for enhanced accuracy. Crystal structures were analyzed by X-ray diffraction (XRD, SmartLab, Rigaku) over a 2θ range of 5–80°, and the obtained patterns were compared with standard JCPDS cards for Au and perovskite phases to confirm the structural identity of the samples. Morphology and microstructure were observed using a field emission scanning electron microscope (FE-SEM, SU8230, Hitachi) and a transmission electron microscope (TEM, Titan ETEM G2). The metal content, including Au loading, was determined using inductively coupled plasma mass spectrometry (ICP-MS, 7700S, Agilent).

**Electrode preparation.** The nanosheet concentration was determined by thermogravimetric analysis (TGA). Gold nanoparticles were deposited on CNO or KCNO supports by a citrate-reduction method. For 11.1 wt% Au loading, 100 mg of support was dispersed in 17.89 mL of DI water and 17.89 mL of ethanol, stirred for 30 min, followed by the addition of 43.68 mL of 0.05 M sodium citrate solution. After 10 min of sonication and 20 min of stirring, 21.74 mL of 0.01 M HAuCl_4_ (42.84 mg of Au) was added under light-shielded conditions and stirred for 20 min. The suspension was centrifuged at 8000 rpm for 5 min, and the collected precipitate was dried at 60 °C for 3 h. Catalysts with 2.62 wt% Au loading were prepared using the same procedure with adjusted volumes of sodium citrate (14.64 mL) and HAuCl_4_ (6.58 mL, 12.97 mg of Au). Catalyst inks were prepared by mixing 8 mg of catalyst (Au/CNO or Au/KCNO) with 1.5 mL of DI water, 0.5 mL of ethanol, and 20 µL of Nafion 117 solution, followed by three 30 min sonication cycles. The overall synthesis procedure of Au/CNO is schematically illustrated in Figure S1. For Au/CNO and Au/KCNO, 436 µL of ink was drop-cast onto carbon paper with a lateral size of 2.8 × 2.8 cm^2^ and dried at 60 °C for 2 h. During CO_2_RR measurements, the exposed area of the working electrode was defined by a circular cell opening with a diameter of 2.0 cm, corresponding to a geometric area of 3.14 cm^2^.

**Electrochemical measurements.** Electrochemical CO_2_ reduction (CO_2_RR) experiments were carried out in a gas diffusion electrode (GDE) cell using a 0.5 M KHCO_3_ aqueous electrolyte saturated with CO_2_. A conventional three-electrode configuration was employed, consisting of a NiFeCuMo foil (thickness: 0.125 mm) as the counter electrode, an Ag/AgCl (3.0 M KCl) reference electrode, and a catalyst-coated carbon paper as the working electrode. All electrodes were connected to a Zive SP2 electrochemical workstation (WonaTech). The cathodic and anodic compartments were separated by a proton-conducting Nafion 117 membrane (Aldrich) to prevent ion crossover. Throughout the electrochemical measurements, high-purity CO_2_ gas was continuously bubbled into the electrolyte at a flow rate of 80 cc min^-1^ to maintain saturation. The catholyte volume was fixed at 40 mL and circulated at a flow rate of 12 mL min^-1^ using a peristaltic pump to ensure uniform mass transport and concentration.

Prior to data acquisition, the working electrode was electrochemically conditioned by applying a constant current of −94.2 mA (corresponding to −30 mA cm^-2^) for 10 min. This conditioning step was performed following previously reported electrode-stabilization procedures to minimize initial transient responses and establish a stable electrode/electrolyte interface before CO_2_RR measurements.^[1,2]^ Linear sweep voltammetry (LSV) was then conducted to evaluate the onset potential and general catalytic behavior under CO_2_-saturated conditions. The potential at which the current density approached zero was used as a reference for subsequent impedance and capacitive measurements.

Potentiostatic electrochemical impedance spectroscopy (PEIS) was employed to assess charge transfer resistance and interfacial kinetics. The uncompensated resistance(R_u_) was obtained from the PEIS measurements, and all electrochemical potentials were iR-corrected according to the following equation:

$$E_{iR-corrected}=E_{measured}-iR_{u}$$

The electrochemical surface area (ECSA) of catalysts was estimated using the double-layer capacitance (C_dl_) method. Cyclic voltammetry (CV) was performed in a non-faradaic potential region at various scan rates. The capacitive current difference was obtained from the anodic and cathodic current densities according to the following equation:

$$\Delta j=j_{a}- j_{c}$$

where $j_{a}$ and $j_{c}$ are the anodic and cathodic current densities, respectively. The $\Delta j$ values were then plotted as a function of scan rate, and the slope of the linear fit was used as a relative indicator of the electrochemically accessible surface area of the catalysts. Because the capacitive current response is proportional to the double-layer capacitance and thus to the ECSA, this slope-based value enables a reliable comparison of the relative ECSA among the catalysts. All samples were analyzed using the same procedure^[3,4]^. Chronopotentiometric (CP) measurements were further carried out under galvanostatic conditions for 1 h at different current densities to evaluate catalytic activity and operational stability during CO_2_RR.

**Product Analysis.** Gaseous products from CO_2_RR were analyzed using gas chromatography (GC, ChroZen GC, Young In) with thermal conductivity detector and flame ionization detectors.

The Faradaic efficiency for gaseous products for CO_2_RR was calculated as

$${FE}_{i}= X_{i} \times v \times\frac{{nFp}_{0}}{{RTi}_{total}}$$

where, $X_{i}$ is the volume fraction of certain product by GC referenced to calibration curves from the standard gas samples, $v$ is the flow rate of gas, $n$ is the number of electrons transferred, $F$ is the Faradaic constant, $p_{0}$ is the standard pressure, $R$ is the gas constant, $T$ is the ambient temperature, and $i_{total}$ is total current. Liquid products were quantified by high performance liquid chromatography (HPLC, ChroZen HPLC, Young In). The Faradaic efficiency for liquid products was calculated as

$${FE}_{i}= Y_{i} \times\frac{{nFp}_{0}}{{RTi}_{total}}$$

where, $Y_{i}$ is the volume fraction of certain product by HPLC referenced to calibration curves from the standard liquid samples.

**Computational details.** Spin-polarized density functional theory (DFT) calculations were performed using the Vienna *Ab* *initio* Simulation Package (VASP).^[5]^ The interactions between ionic cores and valence electrons were described using the projector-augmented wave (PAW) method, and exchange-correlation effects were treated within the generalized gradient approximation (GGA) using the Perdew-Burke-Ernzerhof (PBE) functional.^[6]^ To account for long-range van der Waals interactions, the Grimme DFT-D3 dispersion correction was applied.^[7]^ Electronic self-consistent field (SCF) iterations were converged to 1 × 10^−6^ eV, while ionic relaxations proceeded until the forces on all atoms were less than 1 × 10^−5^ eV/Å. Plane-wave kinetic energy cutoffs of 600 eV and 400 eV were employed for bulk and slab calculations, respectively. Brillouin-zone integrations were carried out using Γ-centered *k*-point meshes appropriate for each supercell size.

The bulk structure of KCa_2_Nb_3_O_10_ (KCNO) was fully optimized using an (8 × 1 × 4) *k*-point mesh, yielding lattice parameters of *a* = 3.89 Å, *b* = 28.89 Å, and *c* = 7.76 Å, which are consistent with experimental values (*a* = 3.88 Å, *b* = 29.51 Å, and *c* = 7.71 Å).^[8]^ Based on the XRD patterns shown in Figures 1h–i, single-layer slab models of the (010) plane were constructed for both KCNO and Ca_2_Nb_3_O_10_ (CNO), as this plane represents the experimentally relevant crystallographic orientation. A (4 × 1) supercell was employed for KCNO (010), while a (2 × 2) supercell was used for CNO (010). The KCNO slab consists of 128 atoms (8 K, 16 Ca, 24 Nb, and 80 O atoms), whereas the CNO slab contains 120 atoms (16 Ca, 24 Nb, and 80 O atoms), as illustrated in Figure S16. The Au/KCNO and Au/CNO interfacial models were built by depositing an Au_5_ cluster onto the optimized KCNO (010) and CNO (010) surfaces, respectively (Figure S17). For all slab calculations, the bottom two atomic layers were fixed at their bulk positions and all remaining layers were fully relaxed. A vacuum spacing of at least 30 Å was introduced along the surface normal direction to prevent interactions between periodically repeated slabs.

The Gibbs free energy change ($\Delta G)$ for each elementary step of CO_2_RR was calculated according to^[9-12]^:

$$\Delta G=\Delta E+\Delta ZPE-T\Delta S$$

where $\Delta E$ is the DFT electronic energy difference, $\Delta ZPE$ is the zero-point energy correction, $\Delta S$ is the entropy change, and *T* is 298 K. Solvation effects were taken into account using the implicit solvent model (VASPsol)^[13]^.

**Supplementary Figures**

**Figure S1.** Schematic diagram of the synthesis of Au/CNO nanosheets through solid-state synthesis, proton exchange, TBAOH-assisted exfoliation, and Au deposition.

**Figure S2.** The TEM images of monolayer CNO nanosheet for thickness analysis.

**Figure S3.** The average sizes of Au nanoparticles for a) Au/KCNO and b) Au/CNO.

**Figure S4.** XPS spectra data of Au/KCNO and Au/CNO powder samples measured without binder, showing (a) Au 4f, (b) K 2p and (c) Nb 3d for Au/KCNO and (d) Au 4f, (e) K 2p and (f) Nb 3d for Au/CNO.

**Figure S5.** The geometric area-normalized current density of a) bare KCNO and CNO support and b) Au loaded KCNO and CNO.

**Figure S6.** Cyclic voltammetry for a) KCNO, b) CNO and Au loaded d) KCNO and e) CNO. Electrochemical surface area (ECSA) of c) KCNO and CNO and f) Au/KCNO and Au/CNO.

**Figure S7.** The faradaic efficiency of wide range potential for a) CNO, b) KCNO c) Au/CNO and d) Au/KCNO.

**Figure S8.** a) TEM images for 2.62 wt% Au loaded CNO. The insets of a) show d-spacing of Au nanoparticle with a 5 nm scale. b) the average sizes of Au nanoparticles for Au_2.62wt%_/CNO.

**Figure S9.** XPS survey data of a) Au 4f, b) K 2p, c) Nb 3d for Au_2.62wt%_/CNO.

**Figure S10.** Cyclic voltammetry for a) Au_2.62 wt%_/CNO. Electrochemical surface area (ECSA) of b) Au_2.62wt%_/CNO.

**Figure S11.** Electrochemical measurements data of Au_2.62wt%_/CNO for a) ECSA normalized current density, b) LSV, c) EIS and d) FE at wide range of potential.

**Figure S12.** The comparison of faradaic efficiency for CNO supported catalysts as Au content increase at wide range potential.

**Figure S13.** Stability evaluation of (a) Au/KCNO and (b) Au/CNO during CO_2_RR. The chronopotentiometric data were recorded for 180 min at approximately −0.85V_RHE_, while the corresponding CO FE were periodically measured during the stability test.

**Figure S14.** XRD patterns of Au/KCNO and Au/CNO electrodes before and after CO_2_RR. Au/KCNO (a) before and (b) after CO_2_RR and Au/CNO (c) before and (d) after CO_2_RR. The purple and yellow reference bars correspond to KCNO (JCPDS No. 98-009-1098) and Au (JCPDS No. 03-065-2870), respectively.

**Figure S15.** SEM images of Au/KCNO (a) before and (b) after CO_2_RR , Au/CNO (c) before and (d) after CO_2_RR.

**Figure S16.** Optimized surface configurations of pristine KCNO (010) and CNO (010) slab models. a) Top view and b) side view of the KCNO (010) surface. c) Top view and d) side view of the CNO (010) surface. The dashed lines indicate the periodic boundaries of the slab models. Color scheme: K (purple), Ca (cyan-blue), Nb (green), and O (red).

 **Figure S17.** Optimized geometries of the Au/KCNO and Au/CNO interfacial models. a) Top view and b) side view of the Au/KCNO interfacial model. c) Top view and d) side view of the Au/CNO interfacial model. The dashed lines indicate the periodic boundaries of the slab models. Color scheme: K (purple), Ca (cyan-blue), Nb (green), O (red), and Au (yellow).

Figure S18. a) Gibbs free energy diagrams for the CO_2_-to-CO reaction pathway on Au(111) and Au/CNO. b) Optimized adsorption configurations of the ^*^COOH and ^*^CO intermediates on Au(111) (top) and Au/CNO (bottom). Color scheme: Ca (blue), Nb (green), Au (yellow), C (brown), O (red), and H (white).

**Figure S19.** The XPS spectra data of O 1s a) before and b) after conversion test for Au/CNO and c) before and d) after conversion test for Au/KCNO.

**Figure S20.** XPS survey data of (a) Au 4f, (b) K 2p, (c) Nb 3d for Au/CNO after conversion test and (d) Au 4f, (e) K 2p, (f) Nb 3d for Au/KCNO after conversion test.

**Table S1.** ICP-MS data for Au loaded catalysts

**Table S2.** XPS-derived peak-area ratios of Au in 2.62 and 11.75 wt% Au/CNO and 10.45 wt% Au/KCNO before and after CO_2_RR.

**■ Reference**

[1] K. Ye, T. Jiang, H. D. Jung, et al., "Molecular level insights on the pulsed electrochemical CO_2_ reduction," *Nat Commun* 15 (2024): 9781, https://doi.org/10.1038/s41467-024-54122-3.

[2] E. L. Clark, J. Resasco, A. Landers, et al., "Standards and Protocols for Data Acquisition and Reporting for Studies of the Electrochemical Reduction of Carbon Dioxide," *ACS Catal* 8 (2018): 6560–6570, https://doi.org/10.1021/acscatal.8b01340.

[3] Z. Jian, J. Yu, I. J. Madatta, et al., "Granular protruded irregular Cu_2_O catalysts for efficient CO_2_ reduction to C_2_ products," *J Colloid Interface Sci* 653 (2023): 1415–1422, 10.1016/j.jcis.2023.09.180.

[4] F. Xi, P. Bogdanoff, K. Harbauer, et al, "Structural Transformation Identification of Sputtered Amorphous MoS_x_ as an Efficient Hydrogen-Evolving Catalyst during Electrochemical Activation," *ACS Catal* 9 (2019): 2368–2380, https://doi.org/10.1021/acscatal.8b04884.

[5] J. Hafner. , "*Ab‐initio* simulations of materials using VASP: Density‐functional theory and beyond," *J Comput Chem* 29 (2008): 2044–2078, <https://doi.org/10.1002/jcc.21057>.

[6] P. E. Blöchl, "Projector augmented-wave method," *Phys Rev B* 50 (1994): 17953–17979, <https://doi.org/10.1103/PhysRevB.50.17953>.

[7] S. Grimme, J. Antony, S. Ehrlich, H. Krieg, "A consistent and accurate *ab initio* parametrization of density functional dispersion correction (DFT-D) for the 94 elements H-Pu," *J Chem Phys* 132 (2010): 154104, <https://doi.org/10.1063/1.3382344>.

[8] H. Fukuoka, T. Isami, S. Yamanaka, "Crystal Structure of a Layered Perovskite Niobate KCa_2_Nb_3_O_10_," *J Solid State Chem* 151 (2000): 40–45, <https://doi.org/10.1006/jssc.2000.8619>.

[9] H. Jeon, H. Kim, J. Lim, H. Shin, "Breaking the linear scaling relationship in CO_2_ reduction by p-block doping at Au(332) surface," *Catal Today* 463 (2026): 115607, <https://www.sciencedirect.com/science/article/pii/S0920586125004250>.

[10] J. Y. Park, M. Ko, G. Park, et al, "Deliberate Construction of IrO_2_ –PdO Heterointerfaces via Sequential Photodeposition for Selective and Durable Chloride Oxidation," *ACS Energy Lett* 11 (2026): 4145–4153, .

[11] E. Jeon, J. Kim, N. Thi Yen Phan, et al., "Competitive oxygen evolution and chlorine evolution on Sb-SnO_2_/IrTaO_x_/Ti electrode: Role of Sb-doping for selective chlorine evolution," 394 (2026): 126848, <https://www.sciencedirect.com/science/article/pii/S0926337326004686>.

[12] K. R. Park, P. M. Nguyen, S. Park, et al., "Fe-Mediated Destabilization of Oxygen Intermediates Boosts Oxygen Evolution in Multimetallic Layered Double Hydroxides," *ACS Appl Mater Interfaces* 18 (2026): 9916–9926, <https://doi.org/10.1021/acsami.5c23304>.

[13] S. M. R. Islam, F. Khezeli, S. Ringe, C. Plaisance, "An implicit electrolyte model for plane wave density functional theory exhibiting nonlinear response and a nonlocal cavity definition," *J Chem Phys* 159 (2023): 234117, <https://doi.org/10.1063/5.0176308>.
